# Supplementary material for: Should I use fixed effects or random effects when I have fewer than five levels of a grouping factor in a mixed-effects model?
Source: PeerJ. 2022 Jan 20;10:e12794. doi: 10.7717/peerj.12794 (PMC8784019; doi:10.7717/peerj.12794)
Supplement: Supplemental Information 1 — The type and number of levels of random effect terms are listed from a survey of the 50 most recent papers in the journal Ecology. Multiple columns indicate multiple random effects mentioned in each publication (each row is one publication). [file peerj-10-12794-s001.docx]

| DOI | GLMM/  LMM | RE1 | RE_N1 | RE2 | RE_N2 | RE3 | RE_N3 |
| --- | --- | --- | --- | --- | --- | --- | --- |
| 10.1002/ecy.3379 | 0 |  |  |  |  |  |  |
| 10.1002/ecy.3377 | 0 |  |  |  |  |  |  |
| 10.1002/ecy.3380 | 1 | sites | 39 |  |  |  |  |
| 10.1002/ecy.3337 | 0 |  |  |  |  |  |  |
| 10.1002/ecy.3385 | 1 | species | 97 |  |  |  |  |
| 10.1002/ecy.3371 | 0 | sites | 20 |  |  |  |  |
| 10.1002/ecy.3381 | 0 |  |  |  |  |  |  |
| 10.1002/ecy.3368 | 1 | species | 18 |  |  |  |  |
| 10.1002/ecy.3363 | 1 | sites | 6 |  |  |  |  |
| 10.1002/ecy.3383 | 0 | individuals | 120 |  |  |  |  |
| 10.1002/ecy.3324 | 0 | populations | 68 |  |  |  |  |
| 10.1002/ecy.3353 | 0 |  |  |  |  |  |  |
| 10.1002/ecy.3356 | 0 |  |  |  |  |  |  |
| 10.1002/ecy.3382 | 1 | ecosystems | 4 |  |  |  |  |
| 10.1002/ecy.3384 | 0 | sites | 53 | years | 61 |  |  |
| 10.1002/ecy.3370 | 1 | publications | 206 | species | ? |  |  |
| 10.1002/ecy.3372 | 0 |  |  |  |  |  |  |
| 10.1002/ecy.3367 | 0 |  |  |  |  |  |  |
| 10.1002/ecy.3376 | 1 | sites | 59 | individuals | ? |  |  |
| 10.1002/ecy.3358 | 0 |  |  |  |  |  |  |
| 10.1002/ecy.3322 | 1 | individuals | 203 |  |  |  |  |
| 10.1002/ecy.3361 | 0 |  |  |  |  |  |  |
| 10.1002/ecy.3364 | 0 |  |  |  |  |  |  |
| 10.1002/ecy.3365 | 0 |  |  |  |  |  |  |
| 10.1002/ecy.3349 | 0 |  |  |  |  |  |  |
| 10.1002/ecy.3362 | 1 | sites | 5 | traps | 50 |  |  |
| 10.1002/ecy.3369 | 0 |  |  |  |  |  |  |
| 10.1002/ecy.3352 | 0 |  |  |  |  |  |  |
| 10.1002/ecy.3344 | 0 |  |  |  |  |  |  |
| 10.1002/ecy.3373 | 1 | populations | 23 |  |  |  |  |
| 10.1002/ecy.3346 | 0 |  |  |  |  |  |  |
| 10.1002/ecy.3354 | 0 |  |  |  |  |  |  |
| 10.1002/ecy.3347 | 0 |  |  |  |  |  |  |
| 10.1002/ecy.3359 | 0 |  |  |  |  |  |  |
| 10.1002/ecy.3360 | 0 |  |  |  |  |  |  |
| 10.1002/ecy.3355 | 1 | years | 10 | blocks | 5 | sites | 6 |
| 10.1002/ecy.3336 | 1 | sites | 21 |  |  |  |  |
| 10.1002/ecy.3366 | 0 |  |  |  |  |  |  |
| 10.1002/ecy.3340 | 1 | sites | 297 |  |  |  |  |
| 10.1002/ecy.3328 | 0 |  |  |  |  |  |  |
| 10.1002/ecy.3345 | 1 | populations | 12 |  |  |  |  |
| 10.1002/ecy.3319 | 1 | individuals | 6 |  |  |  |  |
| 10.1002/ecy.3332 | 1 | experiments/  publications | 39 |  |  |  |  |
| 10.1002/ecy.3321 | 0 |  |  |  |  |  |  |
| 10.1002/ecy.3333 | 0 |  |  |  |  |  |  |
| 10.1002/ecy.3341 | 0 |  |  |  |  |  |  |
| 10.1002/ecy.3334 | 1 | individuals | 40 | individuals | 56 |  |  |
| 10.1002/ecy.3325 | 0 |  |  |  |  |  |  |
| 10.1002/ecy.3315 | 0 |  |  |  |  |  |  |
| 10.1002/ecy.3317 | 1 | species | 251 |  |  |  |  |
