# Supplementary figures and images for: Should I use fixed effects or random effects when I have fewer than five levels of a grouping factor in a mixed-effects model?
Source: PeerJ. 2022 Jan 20;10:e12794. doi: 10.7717/peerj.12794 (PMC8784019; doi:10.7717/peerj.12794)

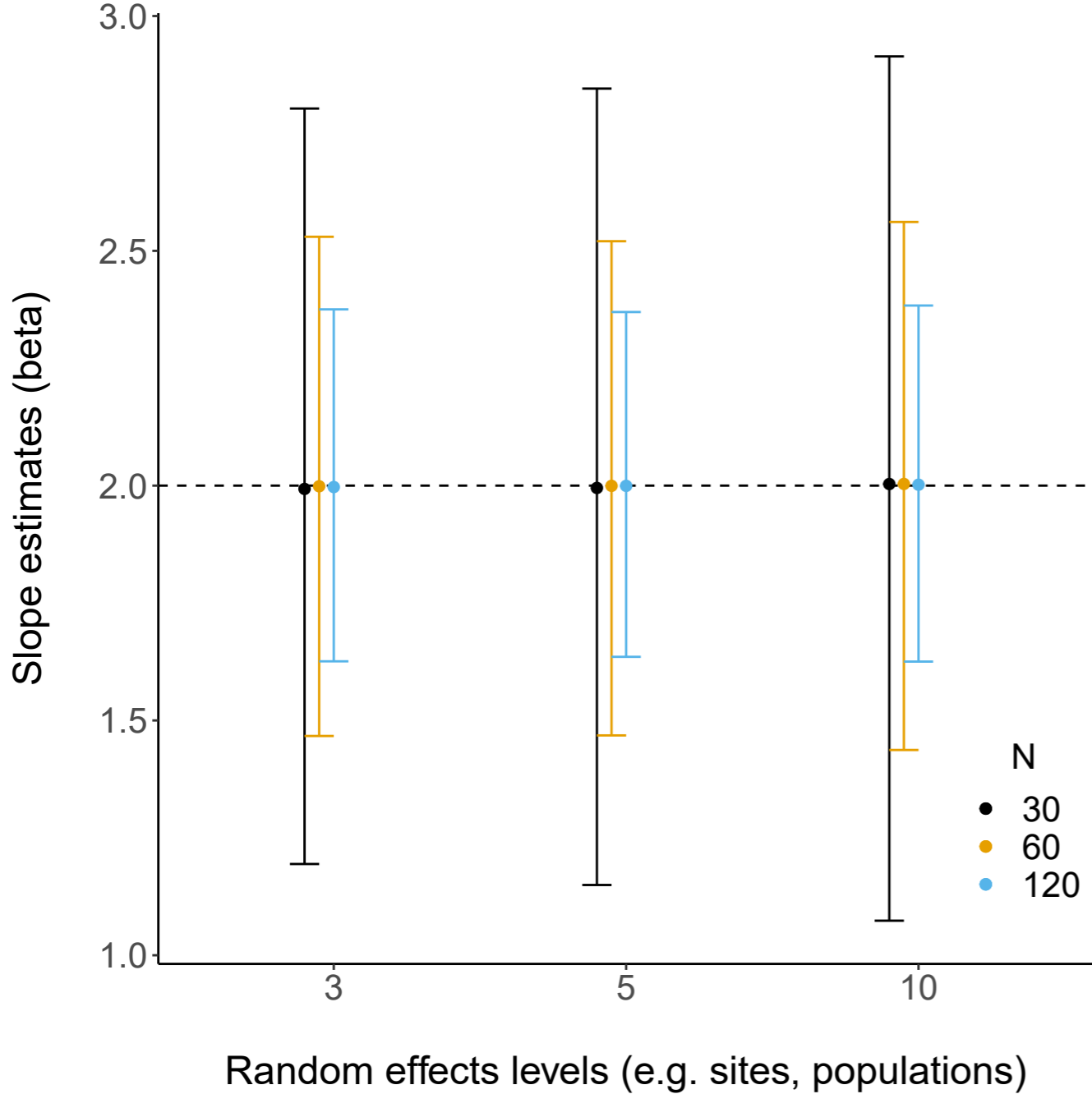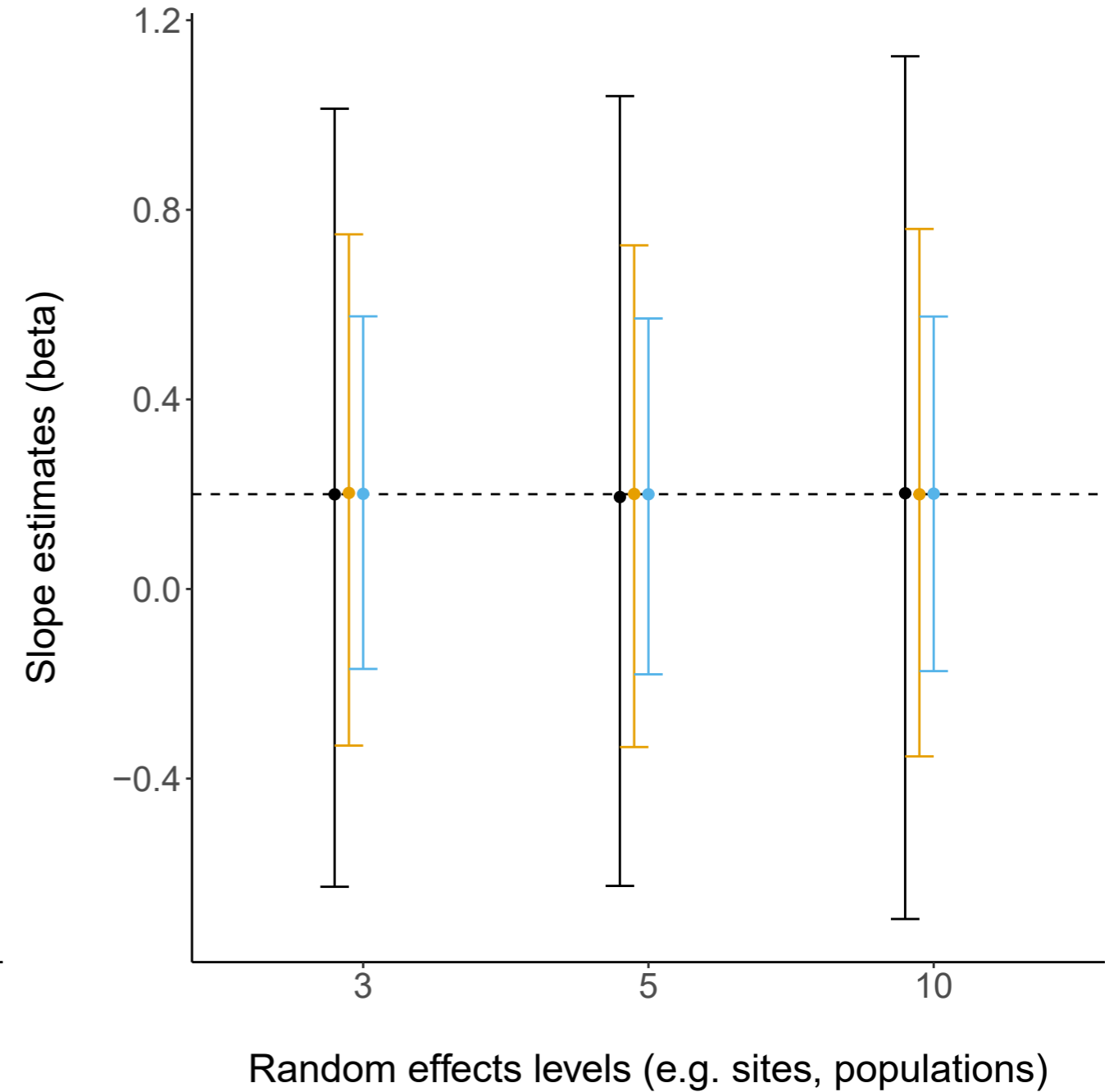

Supplement: Supplemental Information 2 — The stronger slope (true slope = 2; on left) estimates are plotted by the number of levels of the random effect in a linear mixed-effect model. Each point is the mean slope estimate for 10,000 models (and datasets), whereas error bars are 95% intervals (0.025 and 0.975 quartiles) of the distribution of 10,000 point estimates for the slope. N = the number of observations (i.e. number of rows) in each dataset. Dashed lines indicate the true value. The weaker slope (true slope = 0.2) plot (right) is qualitatively similar. [file peerj-10-12794-s002.pdf]

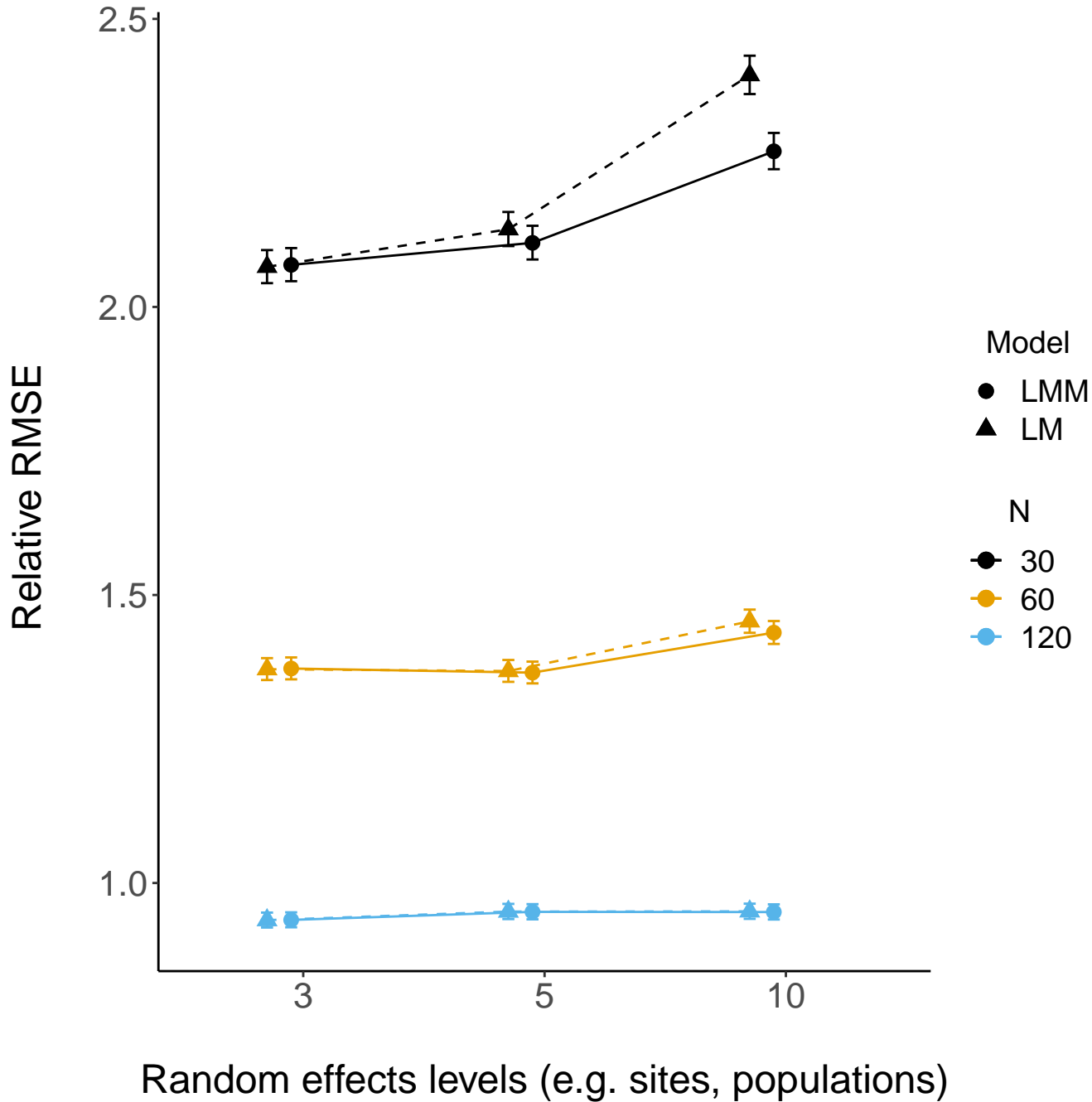

Supplement: Supplemental Information 3 — The relative RMSE of model fixed effects estimates is plotted against the number of levels of a grouping factor. Triangles represent linear model RMSE values (dotted line) and circles represent linear mixed-effect model RMSE values (solid line) for the estimates of slope = 0.2 (see Figure 3 for a plot of slope = 2 estimates), which is qualitatively similar. Colors denote overall sample size (N = 30, 60, or 120). [file peerj-10-12794-s003.pdf]
